# An image of a FGF-23-producing tumor resulting in osteomalacia

**DOI:** 10.1590/2175-8239-JBN-2023-0207en

**Published:** 2024-06-24

**Authors:** Marcelo Giacomini Louça, Ana Laura Mendes, Rodrigo Bueno de Oliveira

**Affiliations:** 1 Universidade Estadual de Campinas, Faculdade de Ciências Médicas, Departamento de Clínica Médica, Divisão de Nefrologia, Campinas, SP, Brazil.; 2 Universidade Estadual de Campinas, Faculdade de Ciências Médicas, Laboratório para o Estudo dos Distúrbios Mineral e Ósseo em Nefrologia (LEMON), Campinas, SP, Brazil.

**Figure 1:**
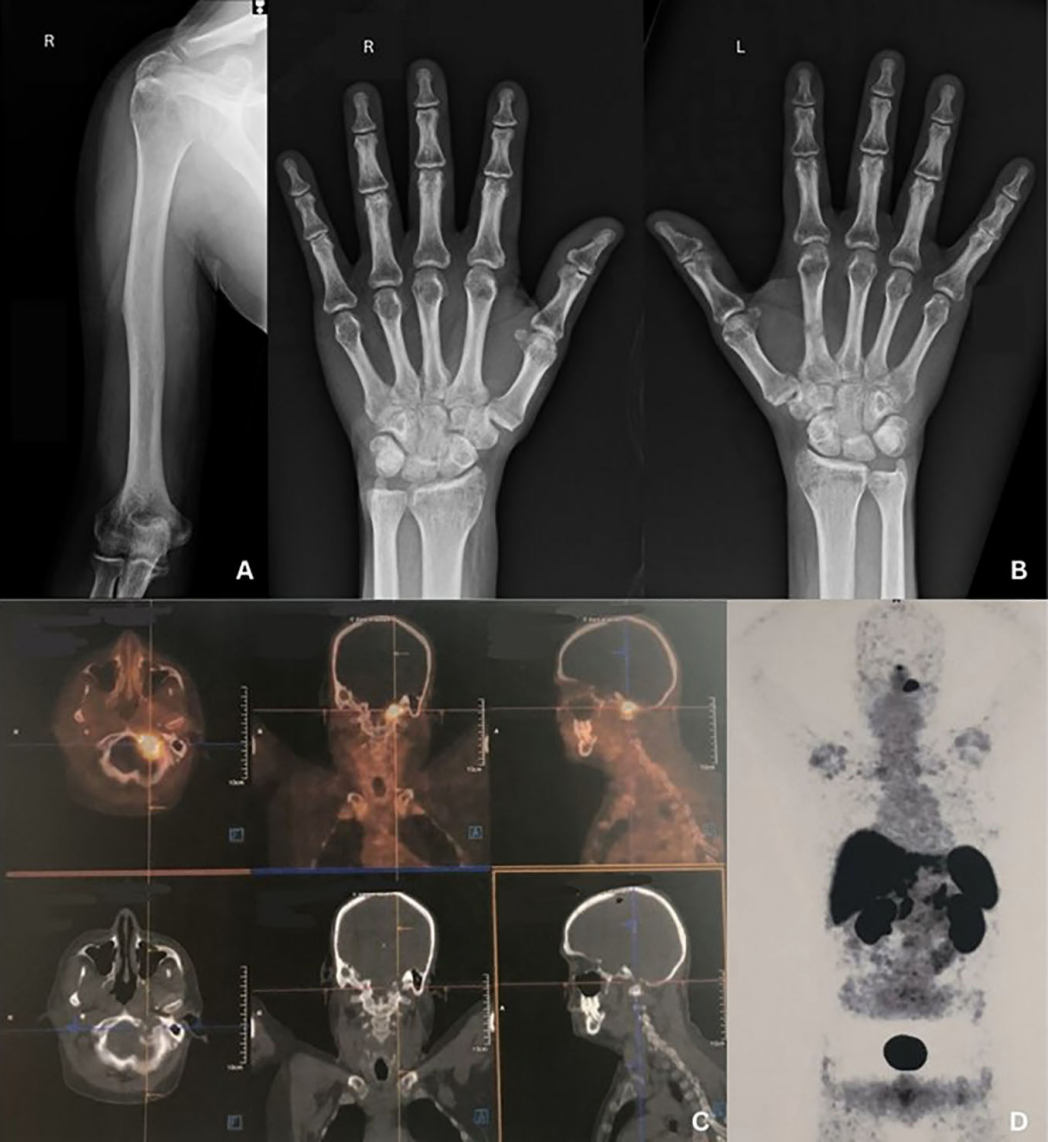
A and B, diffuse bone demineralization and bone deformities suggestive of osteomalacia. C, nodular ^68-^Ga-Dotatate-enhanced image of the left occipital condyle compatible with a tumor-inducing osteomalacia; D, PET-CT showing the location of the tumor and its representation of indirect metabolism.

A 56-year-old male with history of bilateral femoral head replacement due to severe arthrosis sought medical attention due to progression of bone pain and frailty bone fractures. Laboratorial tests revealed persistent hypophosphatemia (0.9–1.6 mg/dL; reference: 2.5 – 4.5 mg/dL) associated to serum FGF-23 of 717 RU/mL (reference: <180 RU/mL), phosphate excretion fraction of 31%, tubular reabsorption of phosphate (TRP) of 69.4% (reference: > 86%), TMP/GFR of 0.62 mg/dL (reference: 2.78 – 4.18), parathormone 80.4 pg/mL (reference: 15 – 65 pg/mL), alkaline-phosphatase 280 IU/L (reference: 43 – 115 IU/L), creatinine 1.03 mg/dL (reference: 0.72 – 1.18 mg/dL), eGFR 109 mL/min/1.73m^2^ (reference: ≥ 90 mL/min/1.73m^2^), and normal total-calcium and 25-vitamin-D levels. There was no personal and family history of rickets or bone diseases. A PET-CT scan labeled with ^68-^Ga-dotatate indicated a mesenchymal FGF-23-producing tumor. The diagnosis based on the images was compatible with a tumor-inducing osteomalacia (Figure 1). The diagnosis of FGF-23-producing tumor and osteomalacia was based on laboratory and imaging findings and the onset of these described clinical manifestations at a late age^1,2,3^.
