# Supplementary material for: An image of a FGF-23-producing tumor resulting in osteomalacia
Source: J Bras Nefrol. 2024 Jun 24;46(4):e20230207. doi: 10.1590/2175-8239-JBN-2023-0207en (PMC11197934; doi:10.1590/2175-8239-JBN-2023-0207en)
Supplement: Translation

# Translation: Imagem de um tumor produtor de FGF-23 resultando em osteomalácia

**DOI:** 10.1590/2175-8239-JBN-2023-0207pt

**Authors:** Marcelo Giacomini Louça, Ana Laura Mendes, Rodrigo Bueno de Oliveira

**Affiliations:** 1 Universidade Estadual de Campinas, Faculdade de Ciências Médicas, Departamento de Clínica Médica, Divisão de Nefrologia, Campinas, SP, Brasil.; 2 Universidade Estadual de Campinas, Faculdade de Ciências Médicas, Laboratório para o Estudo dos Distúrbios Mineral e Ósseo em Nefrologia (LEMON), Campinas, SP, Brasil.

Um homem de 56 anos com histórico de substituição bilateral da cabeça femoral em decorrência de artrose grave procurou atendimento médico devido à progressão da dor óssea e fraturas ósseas por fragilidade. Os exames laboratoriais revelaram hipofosfatemia persistente (0,9–1,6 mg/dL; referência: 2,5 – 4,5 mg/dL) associada a FGF-23 sérico de 717 RU/mL (referência: <180 RU/mL), fração de excreção de fosfato de 31%, reabsorção tubular de fosfato (RTF) de 69,4% (referência: > 86%), TMP/TFG de 0,62 mg/dL (referência: 2,78 – 4,18), paratormônio de 80,4 pg/mL (referência: 15 – 65 pg/mL), fosfatase alcalina de 280 UI/L (referência: 43 – 115 UI/L), creatinina de 1,03 mg/dL (referência: 0,72 – 1,18 mg/dL), TFGe 109 mL/min/1,73m^2^ (referência: ≥ 90 mL/min/1,73m^2^), e níveis normais de cálcio total e 25-vitamina D. Não havia histórico pessoal ou familiar de raquitismo ou doenças ósseas. Um exame PET-CT marcado com dotatato-Ga-^68^ indicou um tumor mesenquimal produtor de FGF-23. O diagnóstico com base nas imagens foi compatível com osteomalácia induzida por tumor (Figura 1). O diagnóstico de tumor produtor de FGF-23 e osteomalácia foi baseado em achados laboratoriais e de imagem, bem como no início das manifestações clínicas descritas em uma idade avançada^1,2,3^.

**Figura 1:** A e B, desmineralização óssea difusa e deformidades ósseas sugestivas de osteomalácia. C, imagem nodular realçada com dotatato-Ga-^68^ do côndilo occipital esquerdo compatível com tumor indutor de osteomalácia; D, PET-CT mostrando a localização do tumor e sua representação indireta do seu metabolismo.
